# Regional Organization of Vacuolar Profiles in the Term Syncytiotrophoblast: A Pilot Ultrastructural Study of a Single Sampling Site in Uncomplicated Pregnancies

**DOI:** 10.3390/biology15211910

**Published:** 2026-10-22

**Authors:** Vanda Rísová, Lívia Gajdošová, Rami Saade, Daniel Kosnáč, Jozef Záhumenský, Štefan Polák

**Affiliations:** 1 Institute of Histology and Embryology, Faculty of Medicine, Comenius University, 813 72 Bratislava, Slovakia; vanda.risova@fmed.uniba.sk (V.R.); rami.saade@fmed.uniba.sk (R.S.); 2 Institute of Medical Chemistry, Biochemistry and Clinical Biochemistry, Faculty of Medicine, Comenius University, 813 72 Bratislava, Slovakia; 3 2nd Department of Gynecology and Obstetrics, University Hospital Bratislava and Comenius University, 826 06 Bratislava, Slovakia; jozef.zahumensky@fmed.uniba.sk; 4 Institute of Medical Physics and Biophysics, Faculty of Medicine, Comenius University, 813 72 Bratislava, Slovakia; daniel.kosnac@fmed.uniba.sk; 5 Institute of Anatomy, Faculty of Medicine, Comenius University, 813 72 Bratislava, Slovakia; stefan.polak@fmed.uniba.sk

**Keywords:** syncytiotrophoblast, human placenta, transmission electron microscopy, placental barrier, trophoblast vacuolization, vesicular transport, placental ultrastructure

## Abstract

Syncytiotrophoblast (STB) vacuolization is common in term placentas, yet identical-appearing vacuoles are interpreted as physiological or degenerative depending on context, and, to our knowledge, a systematic description of their regional distribution within the STB in a defined cohort of uncomplicated term placentas is lacking. This pilot study aimed to define the regional organization of STB vacuolization in terminal villi within a single, consistently defined sampling site in uncomplicated term pregnancies. Using standardized transmission electron microscopy (TEM) protocols and blinded, independent scoring, we qualitatively evaluated vacuolar profiles in the subapical, perinuclear, and basal STB regions of placentas from ten uncomplicated singleton term pregnancies. Vacuolization followed a consistent, recurrent regional pattern across the cohort—small electron-lucent vesicles beneath the microvillous border, heterogeneous membrane-delimited compartments perinuclearly, and occasional larger vacuoles basally. These membrane-delimited profiles (membrane delimitation being an inclusion criterion) were observed in fields with preserved mitochondria and maintained microvillous architecture, suggesting that the regional context within the syncytial cytoplasm, rather than the mere presence of vacuoles, represents a candidate morphological criterion for distinguishing physiological vesicular activity from nonspecific degeneration, requiring direct validation against pathological comparator tissue. This pilot study is descriptive and requires validation in larger cohorts, pathological comparator groups, and complementary molecular approaches before its conclusions can be generalized; the proposed preliminary descriptive framework may offer a starting point for future studies interpreting trophoblast alterations in placental pathology and for contextualizing emerging environmental exposures, such as microplastic particles, at the maternal–fetal interface.

## Simple Summary

The outer layer of the human placenta, called the syncytiotrophoblast, often contains small fluid-filled compartments known as vacuoles. It has been unclear whether these vacuoles are a normal part of placental function or a sign of cell damage, because their normal distribution within the cell layer has not been systematically described in a defined group of healthy placentas. We examined macroscopically normal placental tissue from a single, consistently sampled site in ten women with healthy, uncomplicated pregnancies using high-resolution electron microscopy and found that these vacuoles exhibit a consistent descriptive regional pattern: small ones consistently appear just beneath the surface facing the mother’s blood, more varied ones cluster around the cell nuclei, and larger ones occasionally appear near the fetal blood vessels. In all cases, these vacuoles occurred alongside otherwise well-preserved cell structures and without obvious signs of cell damage, which is compatible with, but does not prove, normal cell activity. This work provides a preliminary descriptive reference map that, once validated in larger future studies including diseased placentas, may help researchers and pathologists develop and apply criteria for distinguishing healthy placental tissue from tissue affected by disease or by exposure to substances such as microplastics.

## 1. Introduction

The syncytiotrophoblast (STB) constitutes the continuous multinucleated epithelial layer covering the chorionic villi and represents the principal cellular interface between maternal blood and the fetal compartment [1]. Beyond mediating gas and nutrient exchange, the STB orchestrates endocrine activity, immune regulation, and maintenance of the placental barrier [2,3]. Like other polarized transporting epithelia, the STB organizes this activity asymmetrically across its apical-to-basal axis, and ultrastructurally harbors a complex vesicular system—microvillous invaginations, endocytic vesicles, and membrane-bound compartments—engaged in uptake, processing, and transcellular transport [4,5,6].

Transmission electron microscopy (TEM) has underpinned our understanding of the placental barrier since the pioneering ultrastructural studies of human chorionic villi in the 1950s. Boyd and Hughes first delineated the microvillous surface of the STB and its subapical vesicular structures, providing the morphological foundation for subsequent concepts of placental transport [7]. Wislocki and Dempsey (1955) and Enders (1957) subsequently characterized the trophoblastic vesicular system across species, establishing a cross-species structural comparison for placental transport mechanisms [8,9].

A pivotal conceptual shift occurred in 1991, when Jones and Fox fundamentally reinterpreted STB vacuolization not as nonspecific degeneration, but as an integral component of normal placental physiology [4]. They demonstrated that cytoplasmic vacuoles actively participate in micropinocytosis and lysosomal processing, with their formation frequently correlating with physiological dilation of the smooth endoplasmic reticulum (ER) during active steroid synthesis. These observations were later integrated into broader interpretations of STB function, encompassing micropinocytosis, lysosomal processing, and functional remodeling of the syncytial cytoplasm [4,5]. Thus, the presence of vesicular and vacuolar profiles in the STB, including subapical vesicles beneath the microvillous surface [7,8,9], and their involvement in endocytic and transcytotic processes [5,6] are well established. Regional differences within the term trophoblast have also been described, including thin and thick zones of the villous trophoblast with different organelle content [10] and differentiated regions of the cell surface associated with coated vesicles [11]; to our knowledge, however, the apico-basal distribution of membrane-delimited vacuolar profiles within the syncytial cytoplasm has not been assessed systematically across predefined zones in a defined cohort of uncomplicated term placentas.

Despite these advances, the interpretation of STB vacuolization remains context-dependent. In routine morphological assessment, vacuolar profiles are easily confounded with changes associated with edema, cellular stress, or hydropic degeneration when evaluated without reference to surrounding organelles, membrane integrity, and regional syncytial architecture [1,12,13]. Consequently, a rigorously defined description of physiological vacuolar patterns in uncomplicated term placentas is essential to accurately interpret STB ultrastructure in both diagnostic and experimental settings. This ambiguity persists even as placental research increasingly turns to single-cell and spatial transcriptomic approaches, because molecular profiling alone cannot resolve the subcellular membrane architecture that ultimately distinguishes ongoing vesicular trafficking from a breakdown of cytoplasmic integrity; ultrastructural morphology therefore remains the method best suited to directly visualizing this distinction.

A well-characterized descriptive reference may also become relevant for environmental particle research. Reports of micro- and nanoplastics in human placental tissue and experimental evidence that they may interact with or cross the placental barrier [14,15,16] have raised interest in a well-defined reference morphology of the normal trophoblast. If validated in future studies, such a reference might help in interpreting particle-associated findings, whereas in the absence of a defined reference, distinguishing normal vesicular compartments from pathological or exposure-related alterations may be difficult. The present study, however, did not investigate particles or exposure.

The present study therefore aimed to provide a descriptive, region-by-region ultrastructural assessment of STB vacuolar profiles in uncomplicated term human placentas, using predefined syncytial zones and a defined sampling and scoring protocol. The novelty of this work lies in this systematic zonal assessment and not in the presence of vesicular or vacuolar profiles in the STB, which is well established. By mapping the spatial distribution and morphological characteristics of vacuolar profiles in uncomplicated term placentas, this work provides a preliminary descriptive framework that may inform future comparative investigations of placental barrier function, pathological trophoblast alterations, and emerging environmental exposures.

## 2. Materials and Methods

### 2.1. Patient Cohort and Study Design

This prospective observational pilot study included ten women aged 30–40 years with uncomplicated singleton term pregnancies. Participants were enrolled between March 2025 and April 2025 after providing written informed consent.

The study was designed to provide a qualitative, preliminary description of STB vacuolar profiles in uncomplicated term placentas, focusing on the regional organization and morphology of vacuolar compartments in relation to adjacent organelles, the apical microvillous surface, and the basal lamina. Because the inclusion criteria were intentionally restrictive, this description characterizes only this narrowly defined cohort of uncomplicated term pregnancies and should not be assumed to represent the full range of uncomplicated term pregnancies more broadly (Section 4.7).

Inclusion criteria were: (i) singleton pregnancy; (ii) gestational age 39 + 0 to 40 + 6 weeks confirmed by first-trimester crown–rump length measurement; (iii) uncomplicated pregnancy without pre-existing maternal disease, including pre-gestational diabetes, chronic hypertension, or autoimmune disease; (iv) absence of pregnancy complications, including gestational diabetes mellitus (GDM), preeclampsia, fetal growth restriction (FGR), or clinical chorioamnionitis; (v) maternal age 30–40 years; (vi) vaginal delivery or cesarean section without intrapartum complications; and (vii) an appropriate-for-gestational-age neonate, defined as birth weight between the 10th and 90th percentile.

Exclusion criteria were: (i) multiple pregnancy; (ii) maternal smoking or substance abuse during pregnancy; (iii) medication affecting placental function, including anticoagulants, antihypertensive therapy, or corticosteroids; (iv) placental abruption or placenta previa; (v) fetal structural or chromosomal anomaly; (vi) meconium-stained amniotic fluid; (vii) rupture of membranes lasting more than 24 h; and (viii) clinical signs of chorioamnionitis or fetal infection.

Placental eligibility was determined by these clinical and macroscopic criteria; formal placental histopathological examination was not performed, and “uncomplicated pregnancy” therefore reflects clinical rather than histological normality (see Section 4.7).

The cohort comprised eight vaginal deliveries and two cesarean deliveries (mode of delivery for each placenta is given in Table 1); the indications for cesarean delivery were breech presentation in one case and previous cesarean section with maternal request for repeat cesarean delivery in the other. Other intrapartum variables potentially relevant to placental ultrastructure, including labor induction, labor duration, oxytocin exposure, anesthesia, and duration of membrane rupture, were not systematically extracted for this pilot study and are therefore not reported (Section 4.7). Placental tissue was sampled within 10 min of placental delivery. Mean maternal age was 34.3 years, mean pre-pregnancy body mass index was 22.4 kg/m^2^, and mean gestational age at delivery was 39.7 weeks. Neonatal birth weight ranged from 3150 to 3680 g (mean 3420 g), and all neonates had 5 min Apgar scores ≥9. Six neonates were female and four were male (Table 1).

### 2.2. Tissue Sampling and Fixation

Placental tissue samples were obtained from the central region of the placental disc, approximately 2–3 cm from the umbilical cord insertion, while avoiding marginal areas within 2 cm of the placental edge. Only macroscopically normal parenchyma was collected, with careful exclusion of areas showing visible infarction, hemorrhage, calcification, or extensive fibrin deposition, based on gross visual inspection at the time of sampling; no formal histopathological assessment of the placenta was undertaken. Sampling was performed from a single site per placenta, representing one cotyledon; cord insertion type (central versus eccentric) and consistency of maternal- and fetal-surface orientation relative to the sampling site were not systematically recorded across the cohort. This single-site sampling strategy was chosen to allow standardized, high-resolution ultrastructural comparison across placentas within the scope of this pilot study, but it does not capture the known regional heterogeneity of the placenta (Section 4.7). Because areas showing visible infarction, hemorrhage, calcification, or extensive fibrin deposition were deliberately excluded, the sampled tissue represents selected macroscopically normal parenchyma rather than a random or complete cross-section of each placenta; this distinction is maintained throughout the manuscript whenever the findings are described as a “normal” or “uncomplicated” placental morphology. For each placenta, the two tissue blocks processed for TEM (Section 2.4) were both obtained from this same predefined central region at a consistent anatomical position relative to the cord insertion, following an identical selection procedure across all ten placentas; block selection was therefore standardized and not left to ad hoc judgment at the time of sampling. Samples were immediately immersed in fixative (within 5 min of collection) and fixed in 2.5% glutaraldehyde (Merck, Darmstadt, Germany; Cat. No. 1.04239) in 0.1 M phosphate buffer (pH 7.3) (NaH_2_PO_4_·H_2_O, Merck, Darmstadt, Germany, Cat. No. 1.06346; Na_2_HPO_4_·7H_2_O, Merck, Darmstadt, Germany, Cat. No. 1.06575) at 4 °C for 2 h using a tissue-to-fixative ratio of approximately 1:10. Following primary fixation, samples were washed three times (30 min each) at 4 °C in the same buffer supplemented with 10% sucrose (Sigma-Aldrich, St. Louis, MO, USA; Cat. No. S0389) to maintain osmotic stability. Fixed specimens were stored at 4 °C for 24–48 h before further processing.

### 2.3. Processing for TEM

Post-fixation was carried out in 1% osmium tetroxide (OsO_4_) (Sigma-Aldrich, St. Louis, MO, USA; Cat. No. 75633) in Millonig’s buffer (Delta Microscopies, Mauressac, France; Cat. No. D11582) for 3 h at 4 °C. Following dehydration through a graded ethanol series (Merck, Darmstadt, Germany; Cat. No. 1.00983) (30%, 50%, 70%, 90%, and 100%; 15 min each), specimens were cleared in two changes of propylene oxide (Sigma-Aldrich, St. Louis, MO, USA; Cat. No. 110205) (10 min each) and infiltrated with Durcupan ACM epoxy resin (Sigma-Aldrich, St. Louis, MO, USA; Cat. No. 44611) using a graded protocol: initial infiltration in a 1:1 mixture with propylene oxide overnight, followed by pure Durcupan I (8 h) and Durcupan II (16 h). Polymerization was performed at 60 °C for 72 h.

Semi-thin sections (1 μm) were cut on an RMC MT-7000 ultramicrotome (RMC Boeckeler, Tucson, AZ, USA) and stained with toluidine blue (Merck, Darmstadt, Germany; Cat. No. 1.04172) for light microscopic orientation. This light-microscopic evaluation was also used to prospectively identify terminal villi, recognized by standard morphological criteria including small villous caliber, a dense subepithelial fetal capillary network positioned close to the villous surface, and a thin, attenuated syncytiotrophoblast layer; identification was performed independently by both observers who subsequently conducted the blinded ultrastructural evaluation, and blocks and regions containing terminal villi were selected for ultrathin sectioning on this basis before proceeding to TEM. Ultrathin sections (90–100 nm) were obtained using a diamond knife on a Leica EM UC7 ultramicrotome (Leica Mikrosysteme GmbH, Vienna, Austria) and mounted on 300-mesh copper grids (Micro to Nano BV, Haarlem, The Netherlands; Cat. No. 21-1M0300). By relying exclusively on primary post-fixation with OsO_4_, which preferentially reacts with unsaturated membrane lipids, we obtained a uniform, unstained appearance of vacuolar compartments and intraluminal material. This allowed the relative electron density of luminal contents to be compared across regions and samples under a constant preparation protocol, without interference from differential stain deposition or artifactual precipitation. To mitigate the resulting reduction in lipid bilayer contrast, membrane continuity was assessed exclusively on profiles displaying unequivocal, continuous osmium-mediated osmiophilic boundaries; any profile with ambiguous, discontinuous, or poorly delineated borders was not included in the classification of membrane-delimited profiles (see Section 2.4). Membrane delimitation was thus an inclusion criterion and not an outcome variable. We acknowledge that this conservative approach may underestimate the absolute number of the smallest vesicular profiles, because membrane delimitation was required for inclusion; low-contrast profiles may therefore be underrepresented, which could affect zonal comparisons (see Section 4.7). The unstained protocol was used as the primary approach for analysis and figure selection throughout this study, because avoidance of heavy-metal precipitate artifacts, which can mimic intraluminal content and directly confound interpretation of vacuolar composition, was considered more critical to the aims of this study than the additional contrast that staining would provide. The unstained approach was not formally validated against contrast-stained sections (Section 4.7). A fuller discussion of the trade-offs of omitting post-sectioning heavy-metal staining is provided in Section 4.7. Micrographs were acquired using a Hitachi HT7650 transmission electron microscope (Hitachi High-Technologies Corporation, Tokyo, Japan) operated at 80 kV and equipped with an AMT XR41B CCD camera (Deben UK Ltd., Bury St Edmunds, Suffolk, UK).

### 2.4. Ultrastructural Evaluation

For clarity, the sampling hierarchy used throughout this manuscript is defined as follows, from largest to smallest unit: placenta (the biological sampling unit, *n* = 10); tissue block (two standardized blocks per placenta, Section 2.2); grid (three grids per placenta, sectioned from these blocks); analytical field (a single field of view within a grid, evaluated directly during microscopy; 306 in total); and micrograph (a digital image acquired from a subset of analytical fields for documentation and illustration; 190 in total, not corresponding one-to-one with analytical fields, Section 3.1). All frequencies and percentages reported in this manuscript refer to placentas or analytical fields, as specified at each instance, and not to micrographs. Ultrastructural evaluation was performed on all available grids containing STB from terminal villi, identified prospectively by both observers using light microscopy prior to TEM, as described in Section 2.3. Two tissue blocks per placenta, selected according to the standardized procedure described in Section 2.2, were processed for TEM, yielding 30 grids in total (three grids per placenta), with variable grid yield per block due to uneven section quality. Fields were initially screened at low magnification (×1500–3000) to identify regions suitable for detailed analysis. Grids were examined in a systematic boustrophedon (serpentine) scanning pattern: evaluation of each grid began at the first field in the top-left corner and proceeded row-by-row, alternating direction (left-to-right, then right-to-left on the subsequent row) upon reaching the end of each row, until the entire grid had been examined; this starting point and transition rule were applied consistently across all grids and placentas; grids were examined in a fixed order corresponding to their position on the specimen holder. Every field encountered was evaluated for preservation quality; no fields were selectively skipped based on vacuolar density or apparent morphological interest. Two categories of fields were distinguished:

***Analytical fields***—areas where the STB displayed preserved cellular architecture suitable for ultrastructural analysis, characterized by recognizable mitochondria, intact apical/basal boundaries, and absence of overt mechanical processing artifacts. These fields were used for the qualitative assessment of vacuolar morphology, membrane continuity (assessed on osmium-contrasted profiles), and regional distribution.

***Excluded fields***—areas where severe technical and preparation-induced artifacts (e.g., physical knife chatter, section folds, resin voids, or local extraction holes) physically disrupted the tissue layout or precluded reliable morphological evaluation. To completely avoid circular reasoning regarding tissue integrity, field exclusion was strictly restricted to these mechanical and processing artifacts rather than biological variation or localized cytoplasmic heterogeneity. Any field free of sectioning damage was retained as an analytical field, regardless of its local organelle density or degree of vacuolation. Excluded fields were not included in the qualitative assessment but were documented and counted.

Within analytical fields, the STB was systematically assessed in three predefined regions: the subapical region adjacent to the microvillous surface, the perinuclear region surrounding syncytial nuclei, and the basal region oriented toward the basal lamina and fetal capillary compartment. Because not every analytical field simultaneously contained all three anatomical zones within its section plane, the number of fields in which each specific zone (subapical, perinuclear, or basal) was anatomically represented and evaluable was not separately recorded during the original evaluation. Consequently, the field-level denominator of 306 represents the total number of analytical fields, not the number of fields in which each specific zone was visible and evaluable; this limitation is discussed further in Section 4.7. Each analytical field was evaluated for all three regions where anatomically represented within that field. Zone assignment followed qualitative anatomical criteria: profiles were assigned to the subapical zone when located immediately beneath the microvillous border, to the perinuclear zone when located adjacent to a syncytial nucleus without direct apposition to the microvillous border or basal lamina, and to the basal zone when located adjacent to the basal lamina and fetal capillary compartment. No fixed numerical distance thresholds were applied to delineate zone boundaries; in particular, the perinuclear zone was defined relative to the position of syncytial nuclei rather than by a standardized distance from any anatomical landmark. We acknowledge that this qualitative, landmark-based approach limits the objective reproducibility of zone assignment, particularly in fields where more than one region was represented in close proximity. For a profile to be classified as membrane-delimited, its limiting boundary had to display a continuous, sharply delineated osmiophilic line without gaps, irregularities, or ambiguous transitions into the surrounding cytoplasm. Profiles lacking such unequivocal membrane definition, which may include poorly preserved or degenerative structures, were noted during evaluation but not included in the zonal classification of membrane-delimited vacuolar compartments and were not quantified separately. Membrane delimitation was therefore an inclusion criterion and not an outcome variable. The same applies to the selection of analytical fields on the basis of recognizable mitochondria and intact apical/basal boundaries. Analytical fields were examined in detail at ×6000–15,000. Micrograph acquisition and field screening were performed by one investigator; qualitative evaluation was performed independently by two observers who were blinded to sample identity and clinical data. The classification of vacuolar profiles was compared between observers; discrepant interpretations were resolved by joint re-examination of the micrographs until consensus was reached.

For descriptive summary, the presence or absence of membrane-delimited vacuolar profiles in each predefined zone was recorded per analytical field. The biological sampling unit in this study is the placenta (*n* = 10); the 306 analytical fields evaluated across these ten placentas represent multiple, non-independent observations nested within each specimen, obtained from three grids and two tissue blocks per placenta. Field-level frequencies are therefore reported as descriptive detail characterizing the consistency of a pattern within and across specimens, and do not constitute 306 independent biological replicates. No formal statistical comparison between regions was performed; the analysis presented here is descriptive throughout. Qualitative evaluation and zonal classification were performed directly during microscopy on all analytical fields; a subset of fields was additionally documented by acquired micrographs, with some fields yielding more than one micrograph (e.g., at different magnifications) and others evaluated without a corresponding micrograph being captured. The reported field-level frequencies are therefore based on direct microscopic assessment of all 306 analytical fields, not on the 190 acquired micrographs, which do not correspond one-to-one with analytical fields and served primarily for documentation and figure selection. Whether a given zone was anatomically represented and evaluable in a field, and whether a profile was present within it, were not recorded as separate data fields at the time of scoring; both the absence of the zone and the absence of a profile within an evaluable zone were recorded as a single negative value. Consequently, 306 is used as a common denominator for all three zones throughout this manuscript, and this denominator does not distinguish a true absence of vacuolar profiles from the absence of an evaluable zone within a given field. Agreement was assessed at the level of the analytical field as a whole: a field was counted as concordant only if the two observers’ classifications matched across all anatomically represented zones within that field, and as discrepant if the classification differed in at least one represented zone. This field-level definition corresponds to the same 306 analytical fields reported elsewhere in this manuscript and does not yield a separate zone-level or profile-level agreement statistic. Inter-observer concordance for zonal classification was estimated at 92.2 % (282/306 fields) before consensus resolution. This value represents raw percent agreement rather than a chance-corrected statistic such as Cohen’s kappa; observer-level, per-field classification data required to calculate a chance-corrected agreement statistic were not available for the present analysis, and raw agreement can be inflated when the underlying prevalence of the scored feature is high, as for subapical and perinuclear profiles in this cohort. This figure should therefore be regarded as a descriptive summary measure of concordance before consensus, and not as evidence of validated inter-observer reproducibility. To provide orientational dimensional estimates rather than a formal stereological population estimate, the maximal diameter of unequivocally membrane-delimited profiles was measured in a limited subset of micrographs (*n* = 15 per region). The specific procedure used to select these 15 profiles per region, including whether selection was random, systematic, or based on other criteria, is not further specified in the available methodological record; the reported ranges should therefore be regarded strictly as illustrative morphological measurements and not as representative estimates of the underlying population size distribution.

**Figure 1:**
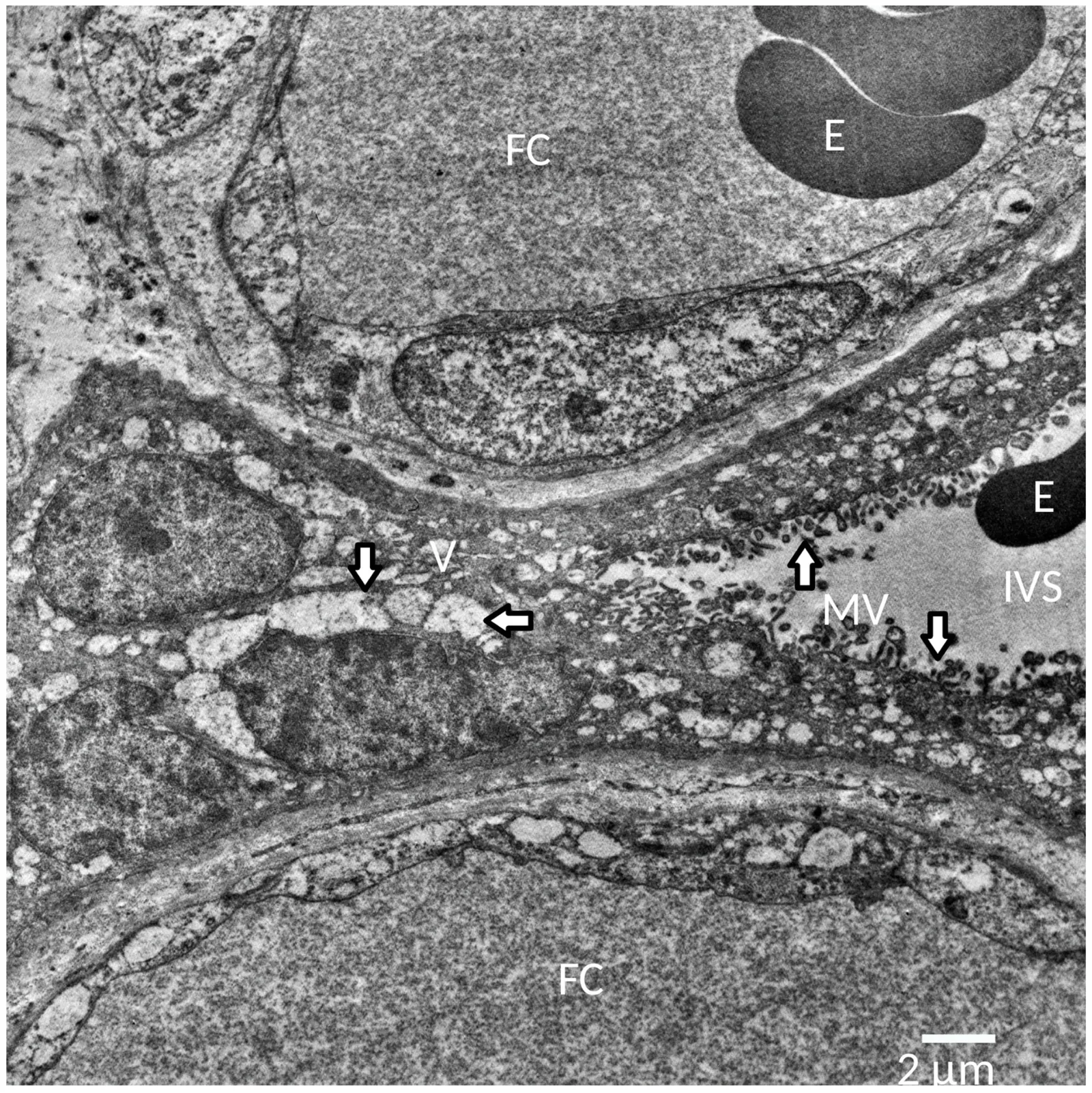
Transmission electron micrograph of the subapical region of the STB in an uncomplicated term human placenta. Numerous small electron-lucent vesicles and larger membrane-delimited vacuolar profiles (V) are located beneath the microvillous border (MV) facing the intervillous space (IVS). Clearly visible fetal capillary (FC) with erythrocytes (E). Arrows indicate representative examples of the corresponding labeled structures. Scale bar: 2 μm.

**Figure 2:**
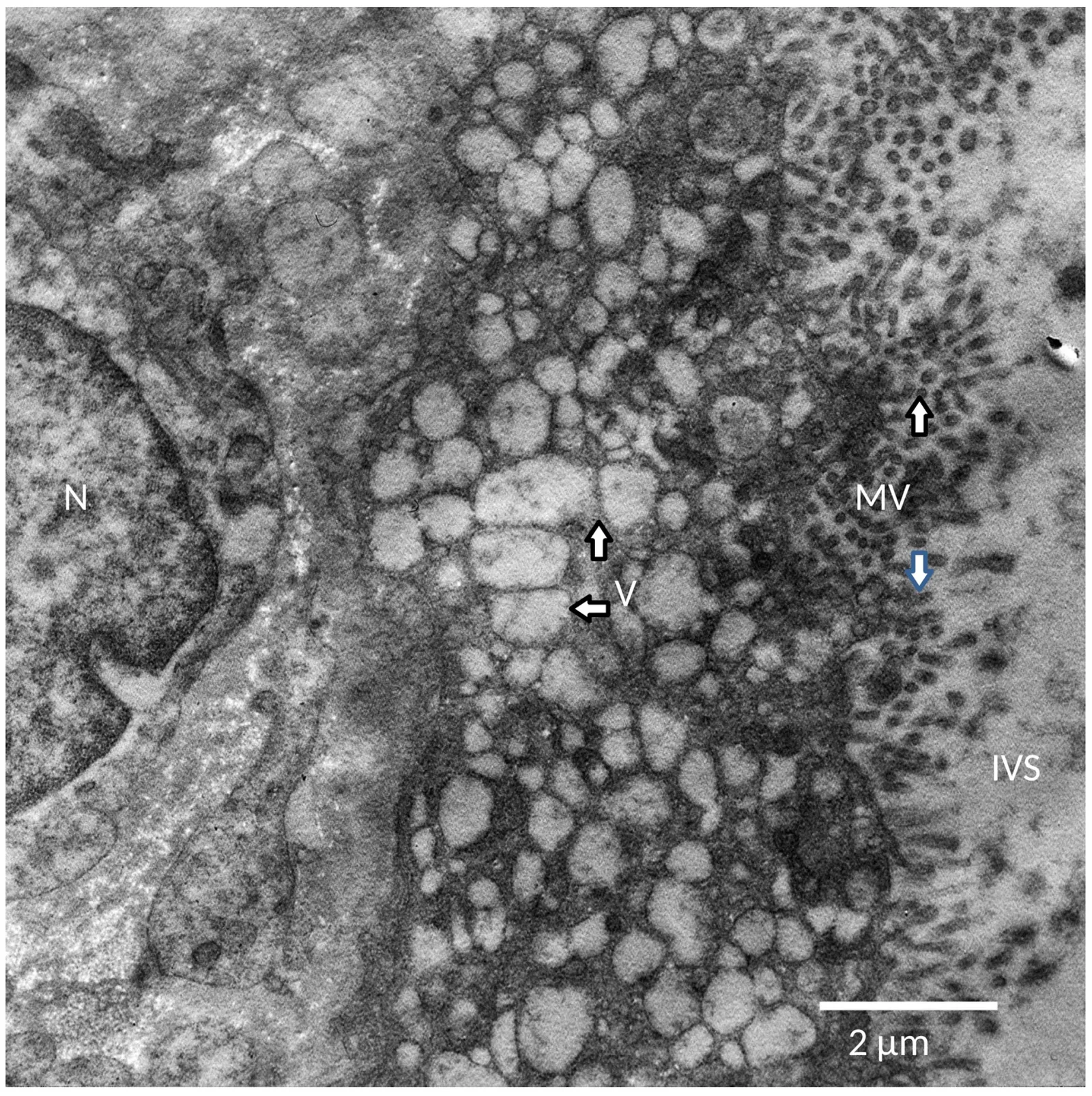
Transmission electron micrograph of the subapical region of the STB in an uncomplicated term human placenta. The subapical cytoplasm is densely occupied by numerous small-to-medium electron-lucent vesicles (V), many of which appear closely apposed; the three-dimensional relationship of these profiles cannot be determined in a single section, and apparent continuity may reflect the plane of section rather than a true shared compartment. Microvilli (MV) face the intervillous space (IVS); a syncytial nucleus (N) is visible at the periphery. Arrows indicate representative examples of the corresponding labeled structures. Scale bar: 2 μm.

**Figure 3:**
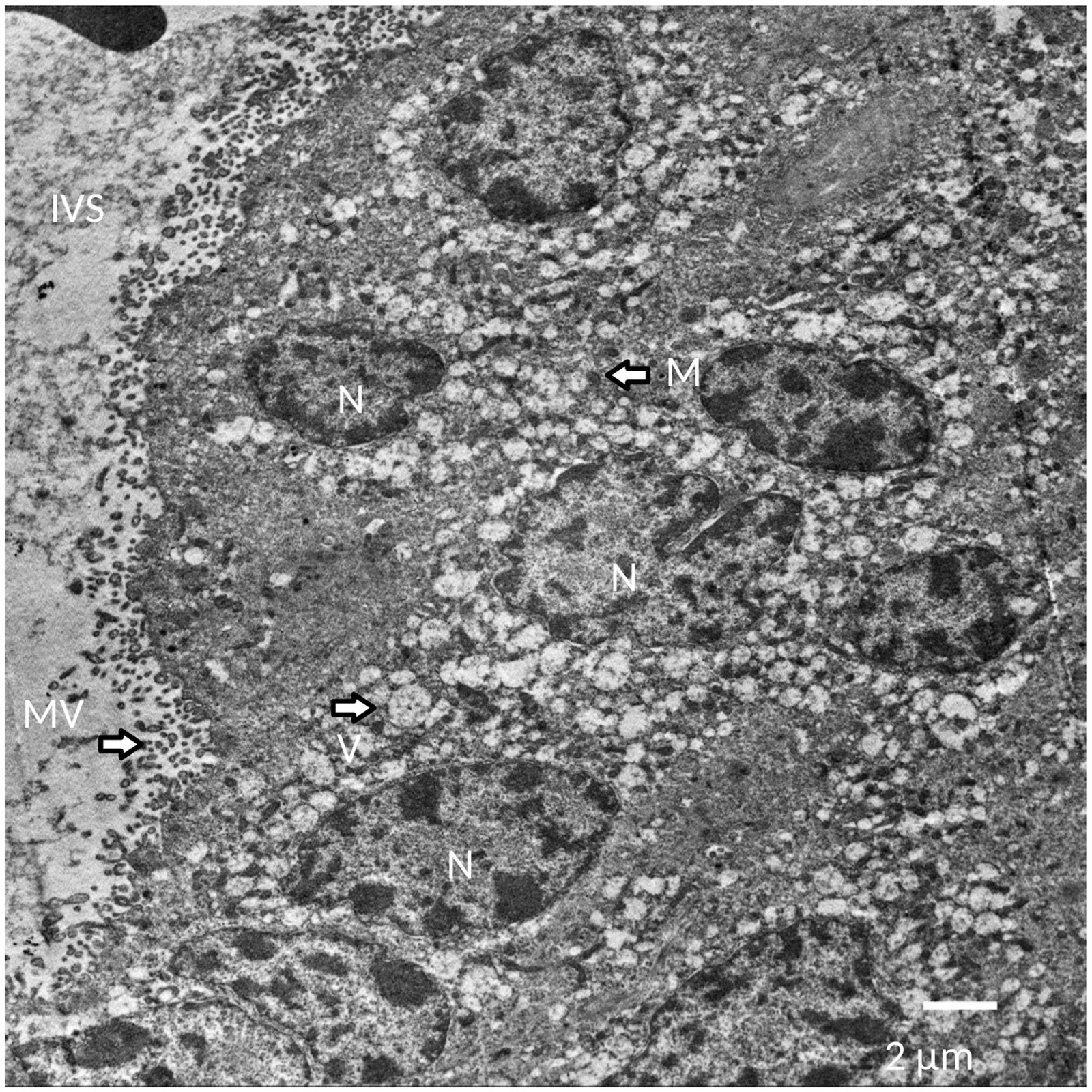
Transmission electron micrograph of the perinuclear region of the STB in an uncomplicated term human placenta. Syncytial nuclei (N) are surrounded by heterogeneous membrane-delimited vacuoles (V) of variable size and electron density; these profiles were classified as perinuclear based on their direct spatial association with the nucleus, irrespective of their distance from the apical surface. Microvilli (MV) at the periphery of the field face the intervillous space (IVS), marking the apical boundary of the same syncytial segment rather than a separate subapical region analyzed here; mitochondria (M) are preserved in the surrounding cytoplasm. Arrows indicate representative examples of the corresponding labeled structures. Scale bar: 2 μm.

**Figure 4:**
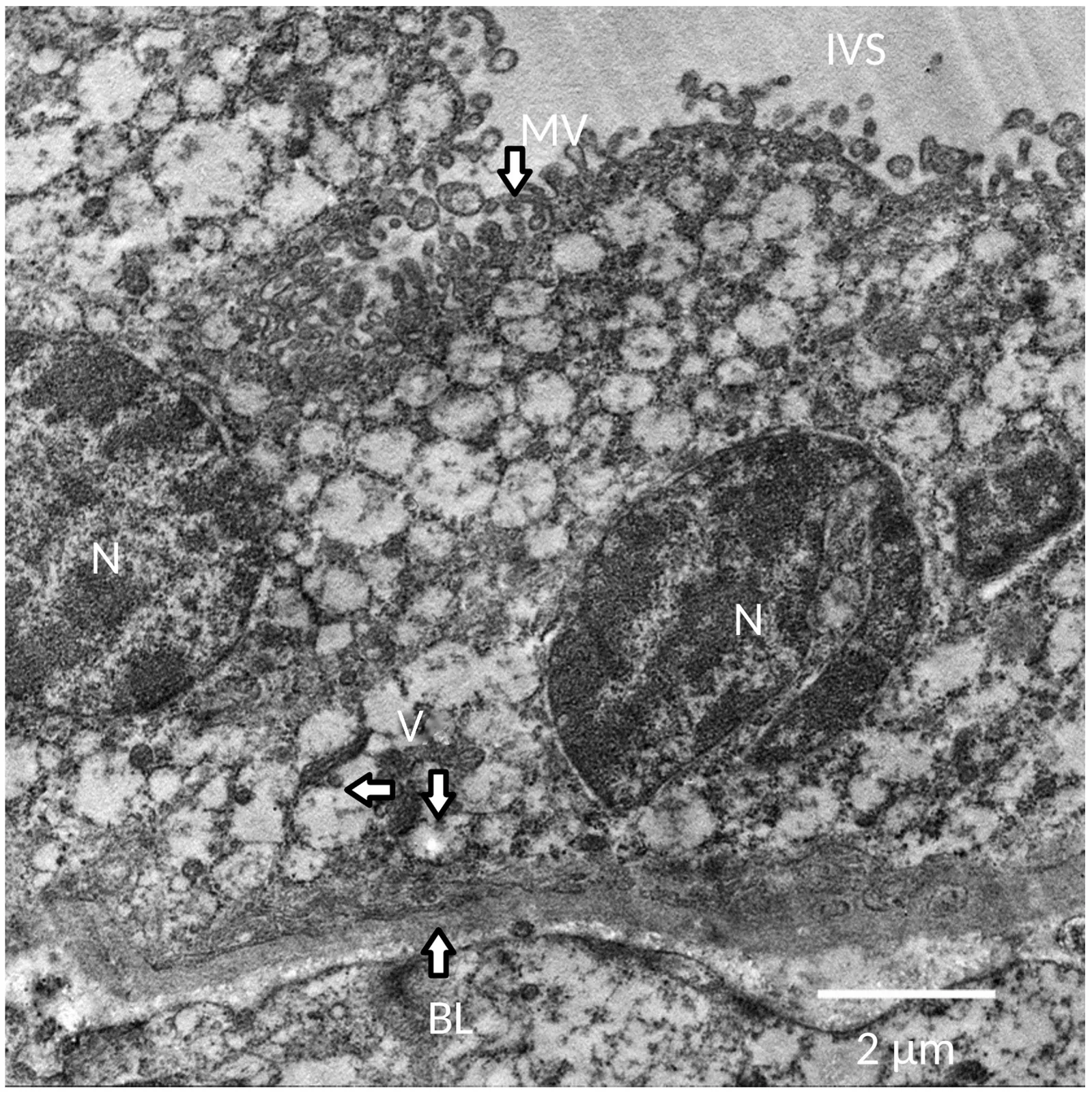
Transmission electron micrograph of the basal region of the STB in an uncomplicated term human placenta. Larger membrane-delimited vacuoles (V) are located near the basal lamina (BL), from which they are separated by preserved cytoplasmic bridges within the characteristically thin basal cytoplasmic rim, distinguishing this region from the thicker perinuclear and subapical compartments visible elsewhere in the same field. A syncytial nucleus (N) shows preserved chromatin organization; microvilli (MV) at the periphery of the field face the intervillous space (IVS), marking the apical aspect of the same syncytial segment. Arrows indicate representative examples of the corresponding labeled structures. Scale bar: 2 μm.

**Figure 5:**
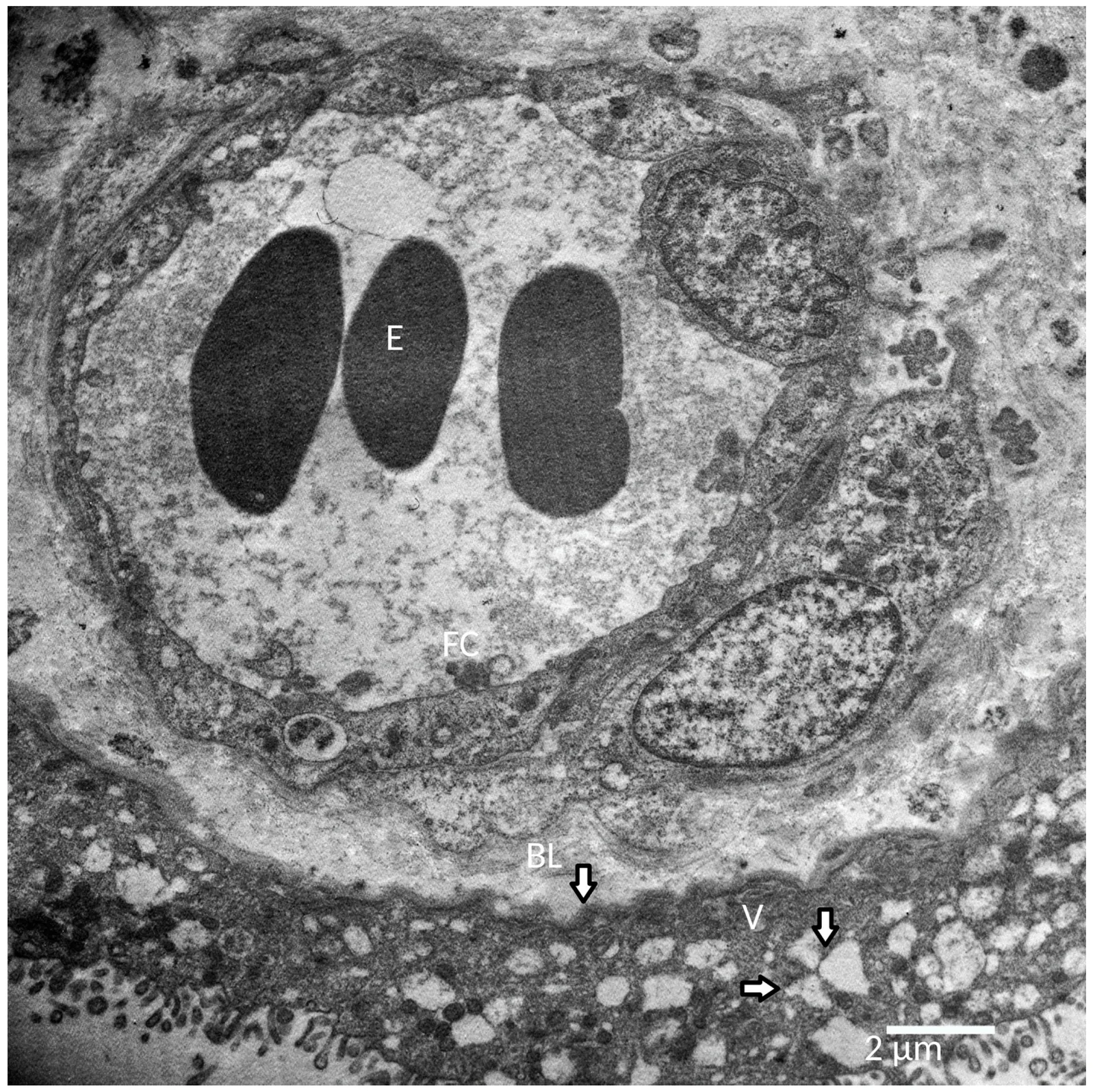
Basal region of the STB. TEM micrograph documenting the distribution of membrane-delimited vacuoles (V) along the basal lamina (BL). The adjacent fetal capillary (FC) lined by intact endothelium contains fetal erythrocytes (E). The profiles are located at the fetal-facing aspect of the STB, and this spatial arrangement is morphologically compatible with transcellular vesicular trafficking toward the fetal capillary, although conventional TEM cannot establish transport direction, functional identity, or trafficking stage of individual profiles. Arrows indicate representative examples of the corresponding labeled structures. Scale bar: 2 μm.

These measurements serve purely as descriptive reference ranges to illustrate relative scale across STB zones. Vacuolar profiles were evaluated qualitatively with respect to size, shape, electron density, membrane delimitation, spatial relationship to adjacent organelles, and regional distribution within the syncytial cytoplasm. For terminological consistency, membrane-delimited profiles were operationally classified as vesicles (maximal diameter ≤ 0.5 μm) or vacuoles (maximal diameter > 0.5 μm). This size-based nomenclature reflects a broadly adopted, but not organ- or cell-type-specific, convention in ultrastructural morphology [4,5], and was applied here strictly as a descriptive classification criterion. It does not imply, and should not be interpreted as evidence for, a discrete biological transition at this diameter, including a change in trafficking stage, luminal content, or functional identity, between profiles above and below the threshold. To our knowledge, no placenta- or syncytiotrophoblast-specific empirical validation of this size boundary has been reported, and the terms ‘vesicle’ and ‘vacuole’ are used throughout this manuscript as morphological size descriptors rather than as designations of distinct subcellular compartments. Moreover, because conventional TEM provides two-dimensional sections (90–100 nm) through three-dimensional structures, the maximal diameter refers to the size of the profile in the section plane and not to the true size or identity of a compartment; the same compartment may appear as a vesicle or a vacuole depending on the plane of section, and a section through an elongated or interconnected structure may appear as an isolated round profile. The analysis focused on recurrent morphological patterns consistently observed across the entire cohort, with particular attention to the spatial organization of vacuolar compartments relative to the apical–basal polarity of the syncytium. Image adjustments, if applied, were limited to global brightness and contrast modifications affecting the entire micrograph equally. No selective enhancement, deletion, or local manipulation of ultrastructural features was performed. Representative micrographs selected for illustration in the main text (Figure 1, Figure 2, Figure 3, Figure 4 and Figure 5) were chosen from the full set of acquired micrographs (*n* = 190) on the basis of technical image quality, specifically optimal focus, absence of sectioning or processing artifacts, tissue continuity, and clear visibility of the relevant subcellular structures, and were intended to depict the pattern most consistently observed within each region across the cohort. Selection was not based on vacuolar density or abundance, and a formal randomized or blinded image-selection procedure was not applied to this illustrative subset. These figures should therefore be understood as technically representative illustrations of the described pattern rather than as an unbiased or randomly sampled subset of the observed morphology, and they do not themselves constitute quantitative evidence of regional prevalence.

## 3. Results

### 3.1. General Preservation of STB Architecture

All analyzed placentas exhibited preserved villous architecture suitable for ultrastructural assessment. Across the ten placentas, a total of 30 grids were examined (three grids per placenta), yielding 360 fields containing STB (Table 2). The 190 micrographs acquired across the cohort (Table 2) do not correspond one-to-one with these fields: some fields yielded more than one micrograph, while others were evaluated directly during microscopy without a corresponding micrograph being acquired. Excluded fields (*n* = 54, 15%) were discarded due to processing artifacts or insufficient cytoplasmic preservation and represented a minor proportion of the total examined material. Exclusion was relatively evenly distributed across placentas, ranging from 5 to 6 excluded fields per placenta (14–17%; Table 2); however, exclusion was not separately tracked by anatomical region (subapical, perinuclear, basal) within excluded fields, so a region-specific exclusion rate cannot be reported (Section 4.7). The STB formed a continuous layer covering the villous surface, characterized by a clearly recognizable microvillous border, preserved cytoplasmic organization, and identifiable basal lamina. Across the evaluated samples, vacuolar profiles were consistently observed within the STB cytoplasm by both observers in all ten placentas. Notably, these profiles showed a consistent descriptive regional pattern in relation to recognizable subcellular regions, including the subapical, perinuclear, and basal compartments.

### 3.2. Regional Organization of Vesicular and Vacuolar Profiles

The STB cytoplasm displayed a consistent spatial pattern in which vacuolar morphology varied systematically with position relative to the apical, perinuclear, and basal compartments, as detailed below for each region. Subapical zone (Figure 1 and Figure 2): The STB formed a continuous layer with a complex apical surface densely covered by microvilli, consistent with preserved apical architecture. The subapical cytoplasm contained numerous small electron-lucent vesicles located immediately beneath the microvillous border. These vesicles were predominantly round to oval; in line with the inclusion criterion, only profiles with a continuous osmiophilic membrane were classified as vesicles (see Section 2.4 and Section 4.7). Their position near the bases of microvilli and the apical plasma membrane is morphologically compatible with vesicular uptake at the maternal-facing surface, but does not by itself demonstrate active uptake, cargo movement, or identify a specific endocytic mechanism. In addition to these smaller vesicles, the cytoplasm contained vacuoles of variable size and shape with electron-lucent content and clear membrane delimitation on osmium-contrasted profiles. Some adjacent vacuolar profiles appeared closely apposed; in a two-dimensional section this may reflect true continuity or fusion of compartments, but also superimposition of separate profiles within the section thickness or sectioning through a single elongated structure, and this ambiguity cannot be resolved without serial-section or volume imaging. Mitochondria were well preserved in the surrounding cytoplasm, and several vacuolar profiles were observed in close proximity to tubular smooth ER. Syncytial nuclei showed irregular ovoid contours with preserved chromatin organization, which is compatible with adequate fixation but does not exclude an artifact introduced uniformly by the processing protocol (Section 4.7). Overall, these findings document prominent subapical vacuolization within preserved STB architecture.

***Perinuclear zone (Figure 3):*** The perinuclear region showed greater morphological heterogeneity of vacuolar profiles compared with the subapical compartment. Vacuoles in this region varied in size and shape, and some contained more electron-dense material or intraluminal membranous structures. Several vacuolar profiles were located close to syncytial nuclei and adjacent granular ER. In the central part of the examined field, multiple membrane-delimited vacuoles occupied a substantial portion of the cytoplasm. Their irregular contours and close spatial apposition may reflect interaction between adjacent vacuolar compartments, but could also result from the plane of section through interconnected or elongated structures. Despite this prominent vacuolization, the surrounding cytoplasm remained preserved, with recognizable organelles and maintained nuclear morphology. These features support the interpretation that perinuclear vacuolization represents an organized ultrastructural pattern within the term STB rather than a nonspecific degenerative change.

***Basal zone (Figure 4 and Figure 5):*** In contrast to the subapical compartment, the basal region showed vacuolar profiles in fewer of the total analytical fields examined (a difference that may partly reflect fields in which the basal region was not represented; Section 2.4), with occasional larger membrane-delimited vacuolar profiles observed near the basal lamina. These vacuoles were separated from the basal lamina by preserved cytoplasmic bridges and mitochondria. In selected areas, vacuolar profiles occupied a substantial portion of the cytoplasm between the nucleus and the fetal-facing basal aspect of the STB. The basal lamina remained clearly recognizable, and the adjacent cytoplasm showed no evidence of diffuse edema or loss of membrane integrity. Syncytial nuclei displayed preserved chromatin organization, and mitochondria remained identifiable in close proximity to vacuolar profiles. These findings indicate that basal vacuolar profiles occurred within a preserved ultrastructural context.

#### Semi-Quantitative Distribution of Vacuolar Profiles

Semi-quantitative assessment showed that vacuolar profiles followed a consistent descriptive regional pattern across the cohort. At the placenta level, the primary biological unit of this study, subapical profiles were identified in 10/10 placentas, perinuclear profiles in 10/10 placentas, and basal profiles in 8/10 placentas. Within these placentas, subapical vesicles were additionally observed in 300/306 (98.0%) analytical fields; perinuclear vacuoles in 288/306 (94.1%) fields; and basal vacuoles in 98/306 (32.0%) fields. Because zone evaluability was not recorded separately from profile presence (Section 2.4), the basal figure in particular may partly reflect fields in which the basal region was not anatomically represented, rather than solely a lower true occurrence of basal vacuoles; the current data do not allow these two possibilities to be distinguished, and this limitation is discussed further in Section 4.7; as noted there, it applies in principle to the placenta-level counts as well, since a placenta in which the basal zone was not evaluable in any sampled field would be indistinguishable from one in which basal vacuoles were genuinely absent. The placenta-level finding—basal profiles present in 8/10 placentas—nonetheless remains the primary summary statistic reported in this study, though it cannot be considered fully independent of this same ascertainment limitation. These field-level percentages describe repeated observations nested within the ten placentas rather than independent biological samples, and are reported here as descriptive detail supplementing the placenta-level findings above. Because profile frequency was not normalized to the cytoplasmic area sampled in each region, and the basal STB compartment is anatomically thinner than the subapical and perinuclear compartments (Section 4.7), the lower basal frequency should be interpreted as a difference in encounter rate within sampled fields rather than as a confirmed difference in true vacuolar density. Initial inter-observer agreement for zonal classification was 282/306 (92.2%) before consensus resolution. Estimated maximal diameters from this limited, non-randomly documented subset ranged from 0.10 to 0.50 μm for subapical vesicles, 0.30 to 1.80 μm for perinuclear vacuoles, and 0.80 to 3.00 μm for basal vacuoles; these ranges are illustrative morphological measurements and should not be interpreted as representative estimates of the population size distribution within each region.

### 3.3. Preservation of Subcellular Structures

A consistent feature across the examined samples was the preservation of subcellular structures in areas exhibiting prominent STB vacuolization. Within the analyzed fields, the observed vacuolization was found alongside an ultrastructurally preserved syncytium, without overt ultrastructural evidence of degenerative change or widespread cellular injury, in this uncomplicated term cohort. As metabolic or functional properties were not measured, this morphological observation does not allow conclusions on the metabolic state of the syncytium. Moreover, because analytical fields were selected on the basis of recognizable mitochondria and intact apical/basal boundaries, this preservation partly reflects the selection criteria and does not constitute an independent finding. It does not, by itself, establish that the vacuolar profiles are physiological; in the absence of a pathological comparator group, this remains a descriptive observation rather than a validated distinction between physiological and degenerative vacuolization. Mitochondria remained identifiable and generally retained recognizable cristae without overt swelling or fragmentation. Syncytial nuclei displayed intact nuclear envelopes and predominantly euchromatic chromatin, without marked chromatin condensation or pyknosis. The ER was preserved in the surrounding cytoplasm, with no extensive cisternal dilation observed in the evaluated fields.

### 3.4. Summary of the Regional Vacuolar Pattern

Across these regions, membrane-delimited vacuolar profiles (an inclusion criterion) were observed in fields with preserved microvillous architecture, recognizable mitochondria, preserved nuclear morphology, and continuous basal lamina. The examined fields showed no evidence of diffuse cytoplasmic edema, widespread mitochondrial swelling, marked chromatin condensation, loss of the apical microvillous border, or disruption of the basal lamina.

These observations suggest that, within the analyzed membrane-delimited profiles and fields, the presence of vacuoles was not accompanied by overt features of degeneration. Because ambiguous profiles and fields lacking preserved architecture were not part of the analysis, this does not exclude that degenerative profiles occur outside the analyzed classification.

## 4. Discussion

### 4.1. Principal Findings and Interpretation of STB Vacuolization

The principal finding of this pilot study is a consistent, recurrent zonal pattern of vacuolar profiles, observed across the examined cohort: the subapical zone contained predominantly small, uniform, electron-lucent profiles; the perinuclear zone exhibited more heterogeneous vacuoles with variable electron density and occasional intraluminal membranous structures; and the basal zone displayed occasional larger vacuoles near the basal lamina. This morphological stratification of profiles in two-dimensional sections is consistent with spatial organization of vacuolar and vesicular profiles within the STB, although this descriptive pattern was not assessed using formal spatial statistics. Such zonation is compatible with the possibility that these profiles reflect distinct, region-specific vesicular processes tied to the STB’s known apical–basal functional polarity, rather than a single, spatially uniform phenomenon; however, this interpretation remains a morphological hypothesis, since conventional TEM cannot establish it and the size-based vesicle/vacuole distinction used here does not itself establish separate functional identities across regions. We emphasize that, because zone evaluability was not recorded separately from profile presence, the lower basal prevalence may partly reflect fields in which the basal region was not represented rather than a confirmed true difference in vacuolar occurrence between zones.

The semi-quantitative data describe the consistency of this zonal pattern at the placenta level: subapical and perinuclear profiles were present in all ten placentas, whereas basal profiles were present in eight of ten. Within placentas, subapical and perinuclear profiles were additionally observed in >94 % of analytical fields, whereas basal vacuoles were observed in approximately one-third of fields; these field-level figures represent repeated, non-independent observations within each placenta rather than additional independent evidence, and are presented as descriptive detail. This gradient-like distribution is compatible with an organized, region-dependent vesicular architecture, although we did not perform formal nested statistical testing to compare regions, and, because profile frequency was not normalized to cytoplasmic area, sampling geometry, particularly the comparatively thin basal STB compartment and the fact that the basal region was not represented in every field (zone evaluability was not recorded), may account for part of the observed basal-to-apical difference (Section 4.7). In addition, as conventional two-dimensional TEM does not reveal the three-dimensional organization of the compartments, this distribution of profiles cannot by itself exclude other interpretations (Section 4.7). We also note that, because an identical fixation and processing protocol was applied across all specimens, the consistency of this pattern across all ten placentas cannot by itself exclude a systematic processing artifact as a contributing explanation (Section 4.7).

A second observation is that the analyzed vacuolar profiles occurred in fields with preserved subcellular architecture. Membrane delimitation was an inclusion criterion, and analytical fields were selected on the basis of recognizable mitochondria and intact apical/basal boundaries; these features therefore describe the analyzed material and are not independent evidence of a physiological character. Within these fields, adjacent mitochondria retained recognizable cristae without overt swelling, syncytial nuclei displayed intact envelopes and predominantly euchromatic chromatin, and both the apical microvillous border and the basal lamina remained clearly identifiable. None of the evaluated fields showed diffuse cytoplasmic edema, widespread mitochondrial damage, or loss of surface architecture. The presence of vacuoles alone was therefore insufficient to classify a profile as degenerative; interpretation required the architectural context of the surrounding syncytial cytoplasm.

These observations are in line with the conceptual reinterpretation proposed by Jones and Fox, who argued that STB vacuolization is an integral component of normal placental physiology rather than nonspecific degeneration [4]. They are also consistent with earlier ultrastructural descriptions of subapical vesicular structures in the human chorionic villus [7,8,9] and with the recognized role of endocytic and transcytotic activity in the villous STB [5]. The contribution of the present study is therefore not the description of vacuolization in the term STB, which has been reported previously, but the systematic, region-by-region assessment of the frequency and morphology of membrane-delimited profiles across ten uncomplicated term placentas. At the same time, the findings contrast with routine diagnostic practice, in which trophoblast vacuolization is still frequently read as hydropic or degenerative change when evaluated without reference to surrounding organelles and membrane integrity [1].

A critical prerequisite for identifying true ultrastructural pathology such as hydropic degeneration, hypoxic cell swelling, or vacuolar pathology in preeclampsia and gestational diabetes mellitus (GDM) is a rigorous, unmanipulated description of the healthy morphological pattern. The primary objective of this pilot study was explicitly focused on describing this pattern in strictly uncomplicated term placentas; we do not claim, at this stage, that this description constitutes a validated reference framework, since this would require demonstration of reproducibility, representativeness, and discriminative capacity against pathological cohorts, which were outside the scope of the present pilot study. This descriptive account is further restricted by the narrow demographic and clinical inclusion criteria applied in this pilot cohort (Section 2.1), by the single, consistently defined placental sampling site (Section 2.2), and by the deliberate exclusion of macroscopically abnormal parenchyma (Section 2.2); it should accordingly be interpreted as characterizing selected macroscopically normal tissue from this specific, highly selected population, rather than uncomplicated term pregnancy or the placenta as a whole. Without a robust, standardized mapping of physiological zonal vacuolization under normal conditions, subtle pathological alterations risk being either over-interpreted as degenerative lesions or misclassified altogether. Establishing a preliminary descriptive account of uncomplicated placentas may therefore represent a first step toward future comparative studies on pathological cohorts, although it does not by itself establish validated diagnostic criteria.

An important interpretive point is that the STB is not a passive covering layer of the villous surface, but a specialized epithelial interface with continuous membrane turnover. Its apical surface faces the intervillous space, whereas its basal aspect is oriented toward the villous stroma and the fetal capillary compartment. In this structural setting, the presence of vesicular and vacuolar profiles within the syncytial cytoplasm is not unexpected. What is notable in the present samples is their zonal arrangement and their coexistence with preserved organelles and membrane integrity, a combination that is consistent with organized vesicular activity rather than nonspecific hydropic or degenerative change. We emphasize that this interpretation is based on the absence of degenerative features within an uncomplicated term cohort, and not on direct comparison with pathological placentas; the profiles described here are therefore best characterized as vacuolar profiles consistent with preserved, physiological-type ultrastructural activity, rather than as definitively physiological.

### 4.2. Relationship to Vesicular Transport and Placental Barrier Function

Having described a zonal pattern of STB vacuolar profiles, we next consider how this distribution may relate to the known vesicular activity of the villous STB. The subapical region lies directly beneath the microvillous surface exposed to maternal blood, making it a plausible site of vesicular uptake and early intracellular processing. Endocytic and transcytotic processes in the villous STB have been described as contributors to placental exchange and nutrient transport [5], and more recent work has emphasized placental endocytosis as an important but still incompletely characterized mechanism of maternal–fetal transfer [6].

In the present study, the small electron-lucent vesicles beneath the microvillous border are morphologically compatible with vesicular uptake at the maternal-facing surface, and current work on endocytosis at the maternal–fetal interface supports the relevance of internalization pathways for nutrient handling, immune protection, and pathogen defense [17]. However, conventional TEM morphology alone cannot identify a specific molecular pathway: the observed profiles cannot be assigned with certainty to clathrin-dependent uptake, caveolae-associated mechanisms, lysosomal maturation, or transcytotic carriers without additional markers. The interpretation must therefore remain morphology-based.

The perinuclear pattern requires similar caution. The presence of profiles with variable electron density and occasional intraluminal membranous structures may reflect different stages of intracellular vesicular processing, but conventional two-dimensional TEM cannot reliably distinguish early endosomes, late endosomes, lysosomes, autophagosomes, secretory compartments, or transcytotic carriers. Moreover, in a two-dimensional section an apparent vacuole may represent an isolated compartment, a cross-section through an elongated structure, interconnected compartments, or part of a larger network, so the diversity of profiles does not necessarily correspond to distinct compartment types. We emphasize that the operational vesicle/vacuole size threshold defined in Section 2.4 is not intended to map onto any of these specific compartments, and profiles on either side of the 0.5 μm boundary should not be assumed to represent sequential stages of a single trafficking pathway without molecular validation. Such a distinction would require immunolabeling, correlative imaging, or molecular markers of specific vesicular pathways.

Several molecular pathways may nevertheless be relevant for the future characterization of these vacuolar populations. Clathrin-dependent and clathrin-independent uptake pathways, caveolae-associated mechanisms, and lipid microdomain-related processes have all been discussed in the context of placental endocytosis [6], and flotillin expression has been investigated in the human placenta as a possible contributor to placental transcytosis [18]. These data underscore the need for targeted molecular validation rather than assigning vacuolar profiles to specific transport routes on the basis of morphology alone.

Extracellular vesicle biology is mentioned only as background: STB-derived extracellular vesicles mediate maternal–placental communication [19,20,21,22]. The present study examined intracellular profiles by conventional TEM and provides no evidence on extracellular vesicle biogenesis or release.

Taken together, the observed zonal vacuolar pattern is morphologically compatible with an organized vesicular architecture within the term STB. The findings may be consistent with known vesicle-associated functions of the placental barrier, but they do not demonstrate a specific function, transport pathway, or the molecular identity of individual vacuolar compartments.

### 4.3. Interpretive Framework for Distinguishing Preserved from Degenerative Vacuolar Morphology

One practical implication of this study is that STB vacuolization should not be evaluated as an isolated finding. A membrane-delimited vacuole surrounded by preserved mitochondria, intact cytoplasmic architecture, euchromatic nuclei, and a maintained microvillous border has a different implication from a poorly delimited vacuolar space occurring within edematous or disrupted cytoplasm. This distinction is particularly important in term placentas, where physiological maturation and pathological injury may share superficially similar morphological appearances. For the practicing perinatal pathologist, this distinction could in the future influence whether a vacuolated STB is read as an incidental feature or flagged as evidence of placental insufficiency; whether the present observations can support such a distinction remains to be tested.

The descriptive observations reported here may serve as a starting point for such a differential interpretation, which was not tested in the present study. In the present samples, vacuolar profiles consistent with preserved ultrastructural activity were, by the inclusion criterion, membrane-delimited profiles with continuous limiting membranes, and they occurred adjacent to preserved organelles. We note that this characterization describes the morphological features observed in an uncomplicated cohort and, because membrane delimitation was an inclusion criterion, it does not in itself constitute proof that these profiles are physiological; such a claim would require direct comparison with pathological placentas, which was beyond the scope of this pilot study. In placentas from preeclampsia, GDM, or fetal growth restriction (FGR), vacuolar change has been described in association with mitochondrial swelling, ER dilation, or autophagic compartments [23,24,25,26,27]; as such placentas were not examined here, the present observations cannot be compared with them.

Pathological placental injury is frequently discussed in relation to oxidative stress (OS), which may affect cellular membranes, proteins, lipids, and DNA [12,23]. Mitochondrial dysfunction is also a recurrent theme in placental disorders, particularly preeclampsia [24,25]. Disturbance of ER homeostasis and altered autophagy have likewise been linked to placental dysfunction and adverse pregnancy states [26,27]. These mechanisms provide the background against which vacuolar change should be interpreted.

In the present samples, several features argued against nonspecific hydropic or degenerative change. These preserved features (Section 3.3) support the interpretation that the observed membrane-delimited vacuolization occurred within preserved STB ultrastructure; this interpretation does not extend to ambiguous or degenerative profiles outside the classification.

### 4.4. Potential Future Applications in Placental Pathology

The preliminary descriptive framework proposed here may, once validated against pathological placentas, be useful for future studies interpreting vacuolization in conditions such as preeclampsia, GDM, and FGR; at present, however, it represents an unvalidated starting point rather than an established diagnostic reference. In preeclampsia, ultrastructural and functional abnormalities have been associated with mitochondrial dysfunction, trophoblast injury, apoptosis, basement membrane changes, OS, and altered release of STB-derived extracellular vesicles [23,24,25,28,29,30,31]. Ceramide-induced lysosomal biogenesis and exocytosis have also been implicated in exosomal release related to endothelial dysfunction in early-onset preeclampsia [32]. These pathological mechanisms, including the altered release of extracellular vesicles, were not investigated in the present study; they provide a useful contrast to the preserved ultrastructure observed in the present samples (Section 3.3).

Placental alterations in GDM provide another relevant comparison. Histological and ultrastructural studies have described structural changes in placentas from pregnancies complicated by GDM or GDM-related macrosomia [33,34]. Mitochondrial dysfunction in the fetoplacental unit has been proposed as an important component of GDM-associated placental pathology [35]. TXNIP-related mechanisms and ferroptosis-associated oxidative injury have been implicated in GDM-related placental damage [36,37]. In this context, the preserved mitochondrial morphology and maintained STB architecture observed in the analyzed fields of the present cohort did not show ultrastructural features associated with injury; however, metabolic function was not assessed, so this observation does not exclude metabolic alterations.

FGR represents a further pathological context in which vacuolar or stress-related trophoblast changes require careful interpretation. Increased autophagy has been reported in placentas from FGR pregnancies [38], and placental-derived fetal growth restriction has been linked to impaired placental development, altered villous function, and stress-related barrier dysfunction [39]. Histopathological characteristics of placentas from FGR pregnancies have been described in pilot studies [40]. Unlike these pathological settings, the present study examined uncomplicated term placentas and did not identify double-membrane autophagosomal profiles or inclusions of degraded organelles in the evaluated fields.

The structural features described herein, such as organelle co-preservation, zonal segregation, and continuous membrane delimitation (the latter being an inclusion criterion), were identified in uncomplicated term placentas and are not established criteria for distinguishing physiological from pathological vacuolization; whether they discriminate between these conditions is unknown and requires direct, parallel comparison with diseased tissues. Ongoing follow-up work by our group aims to apply the same protocol to placentas affected by preeclampsia, FGR, and GDM to determine whether and how this zonal pattern differs under acute and chronic syncytial stress.

This contextual approach may help prevent overinterpretation of STB vacuolization as a degenerative marker. In pathological placentas, vacuolar change becomes more meaningful when accompanied by organelle damage, cytoplasmic edema, microvillous loss, chromatin condensation, basement membrane alteration, or disruption of the villous barrier. In the absence of such features, vacuolization should be interpreted with caution and in relation to the broader ultrastructural architecture.

The descriptive observations reported here may serve as a preliminary morphological reference for future studies of pathological alterations. Studies comparing this preliminary framework with preeclamptic, GDM, or FGR placentas using standardized TEM protocols will be required to determine whether disease-specific patterns of vacuolar dysregulation exist.

### 4.5. Potential Future Applications in Environmental Exposure Studies

The present study did not investigate microplastics, particle localization, particle uptake, or particle-related trophoblast injury; the following concerns one possible future application only. Microplastics have been reported in human placental tissue [14], and experimental work indicates that nanosized particles can interact with or cross placental barrier models [15,16,41]. Should this be pursued, any particle-related findings would need to be interpreted against a well-defined description of normal STB ultrastructure, so that normal vesicular compartments are not mistaken for exposure-related changes, or vice versa; whether the present descriptive framework can serve this purpose remains to be tested. This study provides no evidence on particle sequestration, detoxification, or transplacental transport.

### 4.6. Future Directions

Volume electron microscopy and serial-section approaches could help clarify whether the vacuolar profiles described here represent isolated or interconnected compartments [42,43]. Comparison with trophoblast organoids and single-nucleus transcriptomic data [44] could help anchor these morphological observations to specific molecular phenotypes, and targeted molecular validation—for example, immunogold labeling or correlative imaging of endosomal, lysosomal, autophagic, or extracellular vesicle markers—would be required to determine the molecular identity of the vacuolar populations observed here.

### 4.7. Limitations

This pilot study has several limitations that should be considered alongside its findings. The sample was limited to ten uncomplicated term placentas from a single center, selected using restrictive inclusion criteria (maternal age 30–40 years, gestational age 39 + 0–40 + 6 weeks, singleton pregnancy, absence of maternal disease or pregnancy complications, and an AGA neonate), and pathological placentas were not examined. Placental selection relied on gross macroscopic normality and clinical criteria for an uncomplicated pregnancy; formal placental histopathological examination was not performed, and subclinical microscopic lesions—such as maternal or fetal vascular malperfusion, villitis, intervillositis, delayed villous maturation, or distal villous hypoplasia—cannot be excluded in this cohort. As a result, the reported zonal pattern characterizes this narrowly defined cohort and should not be equated with a general normative reference for uncomplicated term pregnancy; it remains unknown whether or how this pattern varies with gestational age within the term window, maternal age outside the studied range, BMI, parity, fetal sex and other fetal characteristics, placental characteristics, labor characteristics, or mode of delivery. In particular, the cohort was restricted to a narrow maternal age range of 30–40 years, and the present findings should not be extrapolated to pregnancies in younger or older mothers without further validation. Fetal sex (six female and four male neonates) was not analyzed as a variable; given the known sex-dependent differences in placental structure and function and the small size of the cohort, no sex-stratified comparison was possible, and the reported zonal pattern should not be assumed to be independent of fetal sex. Placental weight and the fetoplacental weight ratio were not systematically recorded and are therefore not reported; these simple parameters, if available in future cohorts, would help further characterize the “uncomplicated” nature of the sample. The reported zonal pattern should therefore be regarded as a preliminary descriptive characterization of this specific population rather than a population-level standard, and the proposed differential criteria for physiological versus pathological vacuolization will require validation in larger, more demographically diverse, and disease-affected cohorts, including preeclampsia, GDM, and FGR. In addition, tissue was sampled from a single, consistently defined site per placenta—the central region of the placental disc, 2–3 cm from the cord insertion—representing one cotyledon; cord insertion type and consistency of maternal/fetal surface orientation were not systematically recorded, and more peripheral or otherwise anatomically distinct regions of the disc were not sampled. Placental villous morphology is known to vary regionally across the disc, and given this well-documented anatomical and functional heterogeneity, the reported zonal pattern should be interpreted as characterizing this specific central parenchymal sampling site rather than the term placenta as a whole; the findings should not be generalized beyond the sampled tissue. Throughout this manuscript, the terms “regional organization” and “zonal pattern” refer to the apical-to-basal axis within the sampled STB rather than to variation across the wider placental surface.

As with any TEM-based study, tissue processing can introduce artifacts such as osmotic swelling, differential shrinkage, or membrane extraction; these were minimized through rapid fixation, osmotic stabilization, and systematic preservation screening. However, because an identical fixation and processing protocol was applied to all ten placentas, a systematic artifact introduced uniformly by this protocol would be expected to reproduce consistently across specimens; the consistency of the zonal pattern across all ten placentas therefore cannot, by itself, be used as evidence against an artifactual origin, and this possibility cannot be fully excluded on the basis of the present dataset alone. Post-sectioning heavy-metal staining with uranyl acetate and lead citrate was prospectively omitted to prevent microprecipitate artifacts within vacuolar lumens (Section 2.3). While primary OsO_4_ fixation reliably delineates lipid membrane boundaries, the overall contrast of non-membranous structures and very small vesicular profiles is intrinsically lower than in conventionally post-stained sections. Because membrane-delimitation was a mandatory inclusion criterion for profile scoring, this approach may preferentially exclude the smallest or lower-contrast profiles. Since the illustrative size ranges differed between zones (Table 3: 0.10–0.50 µm subapical vs. 0.80–3.00 µm basal; derived from a limited, non-representative subset of micrographs), this potential ascertainment bias would not necessarily affect all zones equally and would be expected to affect the subapical zone at least as much as the basal zone; its precise effect on the zonal profile counts cannot be fully quantified from the present dataset. Accepting reduced contrast in exchange for eliminating precipitate-related artifacts relevant to interpreting vacuolar content was considered acceptable for the descriptive aims of this pilot study. However, this unstained approach was not formally validated against post-stained sections for this specific application; future studies should therefore include a systematic, blinded, quantitative comparison between stained and unstained sections across multiple placentas using matched-field scoring.

The classification of vacuolar profiles required a continuous, sharply delineated membrane, and analytical fields were selected on the basis of recognizable organelles and preserved apical/basal boundaries. This may preferentially retain structures that already appear morphologically preserved and under-represent poorly delimited or degenerative profiles. Membrane delimitation and organelle preservation are therefore inclusion criteria and cannot be considered independent evidence that the profiles are physiological. Moreover, preserved organelle morphology describes ultrastructure only; the metabolic or functional state of the syncytium was not assessed. Ambiguous profiles were noted during evaluation but were not quantified separately, so their frequency and possible contribution to the regional pattern cannot be assessed. Systematic recording of ambiguous profiles, together with comparison with pathological placentas, will be needed to test whether these criteria can discriminate physiological from degenerative vacuolization. The cohort included eight vaginal and two cesarean deliveries; with only two cesarean cases, no meaningful comparison between delivery modes was possible, and whether the zonal pattern was qualitatively consistent across both delivery modes was not formally assessed. Labor, uterine contractions, transient intrapartum hypoxia, and the delivery-to-fixation interval may influence syncytial ultrastructure, and this potential source of variability could not be assessed in the present study. The study was based on conventional two-dimensional TEM of 90–100 nm sections, without serial-section, correlative or volume imaging, and did not include immunolabeling or molecular markers. An apparent vesicle or vacuole may represent an isolated compartment, a cross-section through an elongated structure, interconnected compartments, or part of a larger network; profile size and the vesicle/vacuole classification therefore depend on the plane of section, and statements on apposition, continuity or fusion of adjacent profiles are interpretative. The vacuolar profiles also cannot be assigned with certainty to specific endocytic, lysosomal, autophagic, or secretory compartments, and functional interpretation remains morphology-based; statements on the possible functional role of the profiles reflect their morphology and position only and should be regarded as hypotheses. The study also provides no evidence for extracellular vesicle biogenesis or release. Future work incorporating serial-section or volume electron microscopy and targeted molecular validation would help determine the three-dimensional organization and identity of these compartments and refine this preliminary structural description.

In addition, the 306 analytical fields evaluated in this study are nested within, and therefore not independent of, the ten placentas that constitute the biological sampling unit; all frequencies reported at the field level should be interpreted as descriptive detail characterizing within-specimen consistency rather than as independent-sample statistics. No hierarchical or nested statistical model was applied, and future studies intending formal statistical comparison between STB regions should account for this clustered sampling structure. The zonal comparisons in Table 3 are descriptive rather than formally inferentially tested, and the reported size ranges are illustrative measurements rather than a stereological population estimate. The frequency of vacuolar profiles was also not normalized to the cytoplasmic area sampled in each region; because the basal (vasculosyncytial) cytoplasm is characteristically thinner than the subapical and perinuclear compartments, this may partly contribute to the lower prevalence of basal profiles independent of true differences in vesicular activity, and quantitative morphometric normalization in future cohorts would help distinguish these possibilities. A related limitation concerns the denominator used to report field-level prevalence in Table 3. Because the number of analytical fields in which each specific zone was anatomically represented and evaluable was not separately recorded during the original evaluation, the reported field-level percentages use the total number of analytical fields (*N* = 306) as a shared denominator for all three zones. This is a conservative and potentially misleading choice, since a zone that is anatomically absent from many fields will appear less prevalent than a zone represented in nearly every field, independent of any true biological difference in vacuolar density. This concern applies most directly to the basal region, where the reported field-level prevalence of 32.0% may therefore understate the true prevalence of vacuolization within basal STB cytoplasm specifically. This limitation applies, in principle, equally to the placenta-level counts: a placenta in which the basal zone was not evaluable in any sampled field would be indistinguishable from one in which basal vacuoles were genuinely absent. The placenta-level finding—basal profiles identified in 8 of 10 placentas—nonetheless remains the primary summary statistic reported in this study, though it cannot be considered fully independent of this same ascertainment limitation. Future studies should prospectively record, for each analytical field, which of the three zones were anatomically represented, allowing zone-specific denominators to be constructed at both the field and placenta level. A per-placenta, zone-specific breakdown of evaluable fields and vacuole-positive fields, as would ideally accompany the aggregate figures in Table 3, could not be constructed for the same reason: this information was not recorded as a separate data point during the original evaluation, either in aggregate or by individual placenta. We recommend that future studies record this breakdown prospectively to allow direct assessment of pattern consistency across biological specimens.

A further limitation concerns the qualitative, landmark-based definition of the three STB zones. Because zone boundaries were assigned by visual position relative to the microvillous border, syncytial nuclei, and basal lamina rather than by fixed numerical distance thresholds, and because the perinuclear zone in particular was defined relative to nuclear position rather than a standardized distance, zone assignment retains an element of observer judgment that raw percent agreement (Section 2.4) does not fully resolve. A schematic illustration of the operational zone boundaries, and numerically defined boundary criteria, would strengthen the reproducibility of this classification in future studies.

Relatedly, the reported 92.2 % (282/306 fields) inter-observer agreement for zonal classification represents raw percent agreement only. A chance-corrected reliability statistic, such as Cohen’s kappa, could not be calculated because paired observer-level, per-field classification data were not retained for the present analysis. Because raw agreement can be inflated when the prevalence of a scored feature is high, as observed here for subapical and perinuclear profiles, this figure should be interpreted as a descriptive indicator of concordance before consensus resolution rather than as a validated measure of classification reproducibility. Future studies should retain paired observer-level data to allow calculation of a chance-corrected reliability statistic. A further limitation concerns the size measurements reported for each STB zone. Maximal diameters were measured in only 15 membrane-delimited profiles per region, and the specific procedure used to select these profiles, whether random, systematic, or otherwise, cannot be reconstructed from the available methodological record. These measurements should therefore be regarded strictly as illustrative rather than as representative estimates of the true population size distribution within each STB zone, and future studies should apply a predefined, reproducible sampling protocol for morphometric size measurements. The field-level percentages in Table 3 share a single denominator for all three zones. Because the number of analytical fields in which each zone was anatomically represented and evaluable was not recorded separately from the presence of vacuolar profiles, and because micrographs exist for only a subset of the 306 fields, zone-specific denominators cannot be reconstructed retrospectively; the total number of analytical fields (*N* = 306) was therefore used for all three zones. A zone that is absent from many fields will consequently appear less prevalent than a zone represented in nearly every field, independent of any true biological difference; this applies most directly to the basal region, where the reported prevalence of 32.0% may understate the prevalence within evaluable basal cytoplasm. The same limitation applies to the placenta-level counts, since a placenta without basal profiles cannot be distinguished from one in which the basal zone was not evaluable. Future studies should record prospectively, for each analytical field, which zones were anatomically represented and evaluable, so that zone-specific denominators can be reported. A further limitation concerns the characterization of excluded fields. Although the proportion of excluded fields was relatively evenly distributed across the ten placentas (5–6 fields per placenta, 14–17%), exclusion was not tracked according to anatomical region at the time of evaluation, and this breakdown could not be reliably reconstructed retrospectively. Because preservation quality could plausibly differ between STB regions, for example if thinner basal cytoplasm were more susceptible to sectioning artifacts, we cannot fully exclude the possibility that technical exclusion was not uniformly distributed across regions, which could contribute to region-dependent selection bias. Future studies should record exclusion by anatomical region prospectively to address this possibility directly. A related limitation concerns the macroscopic selection of tissue for sampling. Because areas showing visible infarction, hemorrhage, calcification, or extensive fibrin deposition were deliberately excluded during tissue collection (Section 2.2), the material examined in this study represents selected macroscopically normal parenchyma rather than an unselected or complete cross-section of the placenta. References to a ‘normal’ or ‘uncomplicated’ placental morphology throughout this manuscript should therefore be understood as applying specifically to this macroscopically selected tissue. A further limitation concerns the identification of terminal villi. Although terminal villi were identified prospectively by light microscopy using standard morphological criteria, independently by both observers (Section 2.3), this classification was not accompanied by a formally validated, quantitative scoring scheme, and a formal inter-observer agreement statistic for terminal villus identification itself, as opposed to zonal vacuolar classification, was not calculated. Because villous subtype and maturation state can influence trophoblast morphology, residual variability in terminal villus classification cannot be entirely excluded as a source of unmeasured variation in the reported findings. An additional source of unmeasured variability concerns intrapartum factors. Labor induction, labor duration, oxytocin exposure, anesthesia, and duration of membrane rupture were not systematically extracted from the clinical records for this pilot study, although mode of delivery and cesarean indication were recorded (Section 2.1). Given the small sample size (*n* = 10), formal statistical adjustment for these variables would not have been feasible in any case; however, we cannot exclude the possibility that unmeasured intrapartum factors contributed to the observed ultrastructural findings. Future studies with larger cohorts should prospectively record these variables to allow their influence on STB ultrastructure to be assessed. Additionally, the selection of micrographs used for illustration should be noted as a further source of potential presentation bias. Figure 1, Figure 2, Figure 3, Figure 4 and Figure 5 were selected from the full set of acquired images based on technical quality criteria (focus, absence of artifacts, tissue continuity, and clear structural visibility) rather than by a random or blinded procedure. While this selection was not based on vacuolar density or abundance, it remains a form of non-random image selection, and readers should not interpret these figures as a statistically representative or unbiased sample of the observed morphology; quantitative claims regarding regional prevalence rely on the systematic field-level assessment reported in Table 3, not on the illustrative figures. Despite these limitations, the present study offers a structured morphological framework for interpreting STB vacuolization in uncomplicated term placentas. Future work incorporating numerically defined zone boundaries and a schematic reference illustration would further improve the reproducibility of this zonal classification.

## 5. Conclusions

This pilot study shows that, within a narrowly defined cohort of uncomplicated term placentas and specifically in terminal villi from the central villous parenchyma sampled here, syncytiotrophoblast vacuolization is not a uniform or incidental feature, but follows a consistently observed zonal pattern; whether this pattern tracks the cell’s known apical–basal polarity remains a morphological hypothesis requiring functional validation, as noted above. Using conventional two-dimensional TEM, we documented a consistently observed zonal organization of vacuolar profiles in the STB of terminal villi—small electron-lucent vesicular profiles in the subapical region, heterogeneous membrane-bound profiles in the perinuclear region, and occasional larger vacuolar profiles near the basal lamina in fields with preserved organelle architecture and maintained microvillous and basal interfaces; membrane delimitation was an inclusion criterion for the classification of these profiles. Membrane-delimited vacuolar profiles were frequently observed in the subapical and perinuclear regions and less frequently in the basal region of the ten placentas examined. As the frequency of these profiles was not normalized to regional cytoplasmic area, and zone evaluability was not recorded separately from profile presence (Section 4.7), the apparent basal-to-apical gradient should be regarded as a difference in observed frequency, which may partly reflect fields in which the basal region was not represented, rather than a confirmed difference in vacuolar density, pending area-normalized morphometric validation in future studies. These findings are consistent with the hypothesis that STB vacuolar profiles in uncomplicated term placentas reflect organized vesicular activity rather than nonspecific degeneration; however, this remains an interpretation based on preserved ultrastructural architecture in a single, highly selected cohort, and definitive classification as physiological, as opposed to pathological, vacuolization will require direct comparison with pathological placentas and molecular validation in future studies.

The descriptive framework reported here offers a preliminary starting point and candidate morphological criteria for future research aiming to distinguish physiological from pathological vacuolization, and may prove valuable for emerging studies of environmental particle exposure, where normal vesicular compartments must be reliably differentiated from exposure-related alterations. Whether these proposed criteria can reliably discriminate physiological from pathological vacuolization, or normal vesicular compartments from exposure-related alterations, remains to be tested; realizing this potential will require direct validation in larger, more diverse cohorts that include pathological comparator placentas. These observations provide a preliminary morphological framework requiring quantitative, stereological, molecular, and disease-comparator validation.

## Figures and Tables

**Table 1:** Individual clinical and demographic characteristics of the enrolled patient cohort (*n* = 10).

Patient No.	Maternal Age (Years)	Pre-Pregnancy BMI (kg/m^2^)	Gestational Age (Weeks)	Mode of Delivery	Birth Weight (g)	Fetal Sex (F/M)	5 min Apgar Score
1	33	21.8	39 + 0	Vaginal	3460	F	10
2	35	24.2	39 + 4	Vaginal	3680	F	10
3	33	20.5	39 + 2	Vaginal	3360	M	10
4	31	22.0	39 + 5	Vaginal	3200	M	9
5	38	23.5	39 + 3	Vaginal	3550	F	10
6	30	19.8	39 + 6	Vaginal	3150	F	10
7	36	24.5	40 + 0	Vaginal	3620	F	9
8	32	21.2	40 + 1	Vaginal	3300	M	10
9	40	23.1	40 + 2	Cesarean	3480	M	10
10	35	23.4	39 + 2	Cesarean	3400	F	10

**Table 2:** Overview of tissue sampling and ultrastructural evaluation.

Placenta	Delivery Mode	Blocks for TEM	Grids Examined	Fields Containing STB	Analytical Fields	Excluded Fields §	Micrographs Acquired
P1	Vaginal	2	3	38	32	6	21
P2	Vaginal	2	3	36	31	5	19
P3	Vaginal	2	3	37	32	5	20
P4	Vaginal	2	3	35	30	5	18
P5	Vaginal	2	3	36	30	6	19
P6	Vaginal	2	3	37	31	6	20
P7	Vaginal	2	3	35	30	5	18
P8	Vaginal	2	3	36	31	5	19
P9	Cesarean	2	3	35	30	5	18
P10	Cesarean	2	3	35	29	6	18
**Total**	**—**	**20**	**30**	**360**	**306**	**54**	**190**

Analytical fields met the predefined preservation criteria (see Section 2.4); § excluded fields were discarded due to processing artifacts or insufficient cytoplasmic preservation. Note: Excluded fields were not separately classified by anatomical region (subapical, perinuclear, basal); a region-specific exclusion rate is therefore not available.

**Table 3:** Semi-quantitative distribution of vacuolar profiles across STB regions.

STB Region	Placentas with Profiles, *n*/*N* (%)	Analytical Fields with Profiles, *n*/*N* Total Analytical Fields (%) ‡	Illustrative Size Range (µm), Non-Representative †
Subapical	10/10 (100.0)	300/306 (98.0)	0.10–0.50
Perinuclear	10/10 (100.0)	288/306 (94.1)	0.30–1.80
Basal	8/10 (80.0)	98/306 (32.0)	0.80–3.00

† Estimated maximal diameters of unequivocally membrane-delimited profiles from a limited subset of micrographs (*n* = 15 per region), selected by a procedure not fully reconstructable from the available methodological record; reported as an illustrative range rather than a representative population estimate. Note: Size values (*n* = 15 measured profiles per region) are presented exclusively as orientational, illustrative dimensional ranges derived from representative micrographs and do not represent formal stereological morphometry. Maximal profile diameters measured in two-dimensional sections do not correspond to true compartment diameters, which depend on the plane of section. ‡ Analytical-field percentages represent repeated, non-independent observations nested within the ten placentas (biological sampling unit) and are reported as descriptive detail; the placenta-level column (*n*/*N* placentas) constitutes the primary unit of analysis. The denominator (*N* = 306) is the total number of analytical fields, not the number of fields in which each zone was anatomically represented and evaluable, which was not recorded separately (Section 2.4 and Section 4.7). The percentages, particularly for the basal zone, therefore cannot distinguish a true absence of profiles from the absence of an evaluable zone; this limitation applies equally to the placenta-level counts, since a placenta in which the basal zone was not evaluable in any sampled field would be indistinguishable from one in which basal vacuoles were genuinely absent.

## Data Availability

The representative micrographs, raw image datasets, and consensus evaluation data supporting the findings of this study are available from the corresponding author upon reasonable request. All tissue samples and derived ultrastructural images were fully anonymized prior to analysis to ensure complete participant privacy.

## Abbreviations

The following abbreviations are used in this manuscript:

STB	syncytiotrophoblast
TEM	transmission electron microscopy
ER	endoplasmic reticulum
GDM	gestational diabetes mellitus
FGR	fetal growth restriction
OS	oxidative stress
V	vacuolar profiles (vacuoles)
MV	microvilli
IVS	intervillous space
FC	fetal capillary
E	erythrocytes
N	syncytial nucleus
M	mitochondria
BL	basal lamina
EV	extracellular vesicle
vEM	volume electron microscopy
